# Polygamy slows down population divergence in shorebirds

**DOI:** 10.1111/evo.13212

**Published:** 2017-04-10

**Authors:** Josephine D'Urban Jackson, Natalie dos Remedios, Kathryn H. Maher, Sama Zefania, Susan Haig, Sara Oyler‐McCance, Donald Blomqvist, Terry Burke, Michael W. Bruford, Tamás Székely, Clemens Küpper

**Affiliations:** ^1^ Milner Centre for Evolution, Department of Biology and BiochemistryUniversity of BathBathBA2 7AYUnited Kingdom; ^2^ Organisms and Environment Division, Cardiff School of BiosciencesCardiff UniversityCardiffCF10 3AXUnited Kingdom; ^3^ NERC‐Biomolecular Analysis Facility, Department of Animal and Plant SciencesUniversity of SheffieldWestern BankSheffieldS10 2TNUnited Kingdom; ^4^ Institut Supérieur de technologie de Menabe Morondava, Faculty of SciencesUniversity of ToliaraMadagascar; ^5^ U.S. Geological SurveyForest and Rangeland Ecosystem Science CenterCorvallisOregon97331USA; ^6^ U.S. Geological SurveyFort Collins Science CenterFort CollinsColorado80526USA; ^7^ Department of Biological and Environmental SciencesUniversity of GothenburgGothenburg40530Sweden; ^8^ Institute of ZoologyUniversitätsplatz 28010GrazAustria; ^9^ Max Planck Institute for OrnithologyEberhard Gwinner Str.82319SeewiesenGermany

**Keywords:** Dispersal, gene flow, mating systems, migration, sexual selection, shorebird, speciation

## Abstract

Sexual selection may act as a promotor of speciation since divergent mate choice and competition for mates can rapidly lead to reproductive isolation. Alternatively, sexual selection may also retard speciation since polygamous individuals can access additional mates by increased breeding dispersal. High breeding dispersal should hence increase gene flow and reduce diversification in polygamous species. Here, we test how polygamy predicts diversification in shorebirds using genetic differentiation and subspecies richness as proxies for population divergence. Examining microsatellite data from 79 populations in 10 plover species (Genus: *Charadrius*) we found that polygamous species display significantly less genetic structure and weaker isolation‐by‐distance effects than monogamous species. Consistent with this result, a comparative analysis including 136 shorebird species showed significantly fewer subspecies for polygamous than for monogamous species. By contrast, migratory behavior neither predicted genetic differentiation nor subspecies richness. Taken together, our results suggest that dispersal associated with polygamy may facilitate gene flow and limit population divergence. Therefore, intense sexual selection, as occurs in polygamous species, may act as a brake rather than an engine of speciation in shorebirds. We discuss alternative explanations for these results and call for further studies to understand the relationships between sexual selection, dispersal, and diversification.

Sexual selection is often thought of as a facilitator of speciation via female mate preferences leading to prezygotic reproductive isolation (the “engine‐of‐speciation” hypothesis; Morrow et al. 2003). Intense sexual selection can lead to rapid speciation in at least four different ways (Ritchie 2007; Wilkinson and Birge 2010; Gavrilets 2014). First, female preference for males that exhibit particular traits may lead to coevolution between males exhibiting the traits and females preferring the trait either via selection for good genes or sexy sons (Fisher 1930; Lande 1981; Kirkpatrick 1982; West‐Eberhard 1983; Fowler‐Finn and Rodríguez 2016; Ellis and Oakley 2016). Second, negative frequency‐dependent selection on sexually selected traits that are important during intrasexual competition may ultimately result in reproductive isolation (Greene et al. 2000; Seehausen and Schluter 2004; Clutton‐Brock and Huchard 2013). Third, sexual selection might be associated with ecological speciation where sexually selected traits or those involved in sexual communication are under divergent natural selection (Maan and Seehausen 2011; Safran et al. 2013). Fourth, sexually antagonistic coevolution, termed sexual conflict (Parker 1979), between males and females may drive an arms race with male and female (counter) adaptations that lead to exaggerated traits which then form reproductive barriers (Gavrilets 2014).

By contrast, sexual selection may also reduce, instead of amplify, reproductive isolation between populations under some evolutionary scenarios. For example, sexual conflict may enhance interpopulation gene flow if female resistance to pre‐ and postmating manipulation prevents matings in some populations, therefore, promoting the dispersal of local males to find naïve females that have not developed counteradaptations in neighboring populations (Parker and Partridge 1998). In addition, sexual selection could also limit sympatric speciation as assortative mating can reduce the variation that could be selected upon, leading to the fixation of certain traits (Kirkpatrick and Nuismer 2004).

Variance in mating success is typically larger in polygamous than in monogamous species. Polygamous individuals attempt to access as many mates as possible and may need to disperse, especially when breeding is highly synchronized locally, to maximize their reproductive success. Dispersal to increase mate access has been suggested to explain why adults of polygamous and promiscuous birds and mammals travel large distances during the breeding season (Blundell et al. 2002; Woolfenden et al. 2005; Debeffe et al. 2014; García‐Navas et al. 2015; Davidian et al. 2016; Kempenaers and Valcu 2017), whereas monogamous species are often more faithful to breeding sites (Pitelka et al. 1974; Saalfeld and Lanctot 2015). High breeding dispersal is likely to lead to low levels of genetic differentiation within a polygamous species (Küpper et al. 2012; Verkuil et al. 2012; Eberhart‐Phillips et al. 2015). This gene flow may prevent reproductive isolation by counteracting the effect of processes such as genetic drift and local adaptation and thus slowing speciation processes (here termed the “dispersal‐to‐mate” hypothesis).

Regular migration movements outside the breeding season may also influence diversification (Phillimore et al. 2006; Garant et al. 2007; Weeks and Claramunt 2014; Arendt 2015). Intuitively, high dispersal abilities should reduce genetic differentiation between populations (Belliure et al. 2000; Garant et al. 2007; Claramunt et al. 2012; Weeks and Claramunt 2014). Indeed, many examples of low genetic differentiation among breeding populations of migratory species are found in birds and mammals (e.g., Webster et al. 2002; Friesen et al. 2007; Burns and Broders 2014). However, high (and leptokurtic) dispersal can also lead to the colonization of remote areas such as oceanic islands that are too far away from the core population to maintain regular gene flow. After the colonization event, local adaptation, and genetic drift in combination with behavioral changes may then lead to allopatric differentiation (Rosenzweig 1995; Owens et al. 1999; Phillimore et al. 2006). Corroborating this hypothesis, seasonal migration has been linked to greater net diversification rates in birds where colonization events are followed by settling down and loss of annual migratory behavior (Rolland et al. 2014).

Shorebirds (sandpipers, plovers, and allies; *Scolopaci, Thinocori, Chionidi*, and *Charadrii*) are a diverse and ecologically well‐characterized avian clade that display huge variation in mating systems and migratory behavior (Székely et al. 2000; Piersma and Lindström 2004; Thomas et al. 2007; García‐Peña et al. 2009). This group of taxa therefore provide an ideal opportunity to investigate the relationship between mating systems, migratory behavior, and diversification. The objectives of our study were to test whether polygamous species that are under high pressure to access multiple mates, and thus are subject to strong sexual selection, showed higher diversification than monogamous species, as predicted by the “engine‐of‐speciation” hypothesis or lower diversification consistent with the “dispersal‐to‐mate” hypothesis. Mating systems have a significant influence on the variation of individual mate success, with polygamy leading to greater variation in mating success across individuals compared to monogamy (Emlen and Oring 1977; Shuster and Wade 2003). For this reason, we used mating system as a proxy for strength of sexual selection as we hypothesized that due to this high variation in breeding success, polygamous individuals move between breeding populations in an attempt to elevate their chance of successful mating (Breiehagen 1989; Székely and Lessells 1993; Stenzel et al. 1994; Kempenaers and Valcu 2017).

We investigated the relationships between mating systems, migration, and diversification using two datasets with either genetic differentiation or subspecies richness as proxy for within species population divergence and hence speciation propensity. Firstly, we studied plovers (*Charadrius* spp), a globally distributed clade of shorebirds that includes both migrant and resident species with monogamous or sequentially polygamous mating systems (Thomas et al. 2007; dos Remedios et al. 2015). Within a breeding season sequentially polygamous plovers change partners after a successful breeding attempt, leaving their mate to care for the young, whereas monogamous plovers stay together for subsequent breeding attempts. Social mating system reflects genetic mating system in plovers, since extra‐pair paternity is rare in these species (less than 5%, Maher et al. in press). Using ten *Charadrius* species we tested whether intraspecific patterns of genetic differentiation were associated with mating system and/or migratory behavior using microsatellite datasets. Secondly, since similar genetic data are only available for a fraction of species, we expanded our analyses to include 136 shorebird species to test whether mating system and/or migratory behavior predicted subspecies richness, an alternative measure for diversification (Belliure et al. 2000; Phillimore and Owens 2006, Martin and Tewksbury 2008).

## Materials and Methods

### GENETIC DIFFERENTIATION IN PLOVER POPULATIONS

**Figure 1:**
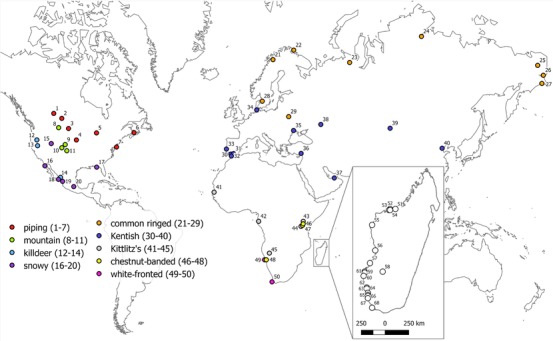
Sampling locations of plover populations for genetic differentiation analyses. Numbers refer to population information (Table 1). In Madagascar insert, symbols do not represent species but rather they show position of sites in North, Middle and South Madagascar.

We analyzed published and newly collected microsatellite data from 10 plover species (Genus: *Charadrius*): Kittlitz's plover (*C. pecuarius*; Eberhart‐Phillips et al. 2015; dos Remedios 2013), Madagascar plover (*C. thoracicus*; Eberhart‐Phillips et al. 2015), white‐fronted plover (*C. marginatus*; Eberhart‐Phillips et al. 2015; dos Remedios 2013), chestnut‐banded plover (*C. pallidus*; dos Remedios 2013), Kentish plover (*C. alexandrinus*; Küpper et al. 2012), mountain plover (*C. montanus*; Oyler‐McCance et al. 2008) and piping plover (*C. melodus*; Miller et al. 2010). In addition, further plover populations from three species were genotyped including, snowy plover (*C. nivosus*), common ringed plover (*C. hiaticula*), and killdeer (*C. vociferous*). Sampling locations were distributed across all continents except Australia, South America, and Antarctica (Table 1, Fig. 1) and included four resident and six migratory species with different mating systems (six monogamous and four polygamous) and wide variation in breeding range sizes (Table 1). The detection of spatial genetic pattern can be highly sensitive to factors such as the number of loci and the number of alleles per locus (Landguth et al. 2012), however, across the datasets we found no relationship between the number of loci or the average number of alleles per locus and the detection of spatial genetic patterns (see Supplementary material). For microsatellite marker characteristics and laboratory protocols see Table S1.

**Table 1:** Summary of sample characteristics for plover species and populations included in genetic differentiation analyses

Species	Subspecies	Population (Map number | Loc Prior)	Latitude, longitude	*N*	Breeding range size (Km^2^)	Mating system	Migratory status
Piping plover	*circumcinctus*	Prairie North (1 | A)	53.2, –110.8	6	221,000	Monogamous	Migratory
*Charadrius melodus*	*circumcinctus*	Prairie South (2 | B)	51.4, –106.0	18			
Miller et al. 2010	*circumcinctus*	Great Plains North (3 | C)	47.6, –102.1	24			
	*circumcinctus*	Great Plains South (4 | D)	42.8, –97.4	23			
	*circumcinctus*	Great Lakes (5 | E)	45.8, –85.6	13			
	*melodus*	Atlantic Canada (6 | F)	45.9, –63.4	66			
	*melodus*	Atlantic USA (7 | G)	39.6, –73.8	70			
Mountain plover	N/A	Northern (8 | A)	47.9, –107.9	21	759,000	Polygamous	Migratory
*Charadrius montanus*		Central (9 | B)	40.8, –104.0	34			
Oyler‐McCance et al. 2008		Montane (10 | C)	39.3, –106.0	15			
		Southern (11 | D)	37.9, –103.1	24			
killdeer plover	*vociferus*	Summer Lake (12 | A)	42.8, –120.8	24	9,100,000	Monogamous	Migratory
*Charadrius vociferous*	*vociferus*	Honey Lake (13 | B)	40.3, –120.3	25			
(this study)	*vociferus*	Ceuta (14 | C)	23.9, –106.9	26			
snowy plover	*nivosus*	Utah (15 | A)	41.2, –112.3	25	1,600,000	Polygamous	Migratory
*Charadrius nivosus*	*nivosus*	San Quintín (16 | B)	30.6, –116.0	22			
(this study)	*nivosus*	Florida (17 | C)	29.9, –85.5	43			
	*nivosus*	Ceuta (18 | D)	23.9, –106.9	25			
	*nivosus*	Nayarit (19 | E)	22.4, –105.6	8			
	*nivosus*	Texcoco (20 | F)	19.5, –99.0	23			
common ringed plover	*tundrae*	Lapland (21 | A)	68.4, 18.5	9	4,530,000	Monogamous	Migratory
*Charadrius hiaticula*	*tundrae*	Varanger (22 | B)	70.3, 30.7	12			
(this study)	*tundrae*	Northern Europe (23 | C)	67.7, 63.6	7			
	*tundrae*	Taimyr (24 | D)	72.9, 105.9	16			
	*tundrae*	North east Chukotka (25 | E)	67.1, –174.5	10			
	*tundrae*	East central Chukotka (26 | F)	64.7, 177.8	11			
	*tundrae*	South east Chukotka (27 | G)	62.5, 177.0	23			
	*hiaticula*	S.Sweden (28 | H)	57.3, 12.1	25			
	*hiaticula*	Belarus (29 | I)	52.1, 27.7	12			
Kentish plover	*alexandrinus*	Doñana (30 | A)	36.4, –6.4	25	13,600,000	Polygamous	Migratory
*Charadrius alexandrinus*	*alexandrinus*	Fuente de Piedra (31 | B)	37.1, –4.8	25			
Küpper et al. 2012	*alexandrinus*	Gharifa (32 | C)	35.2, –6.4	11			
	*alexandrinus*	Samouco (33 | D)	38.7, –8.9	25			
	*alexandrinus*	Beltringharder Koog (34 | E)	54.5, 8.9	13			
	*alexandrinus*	Kujalnik (35 | F)	46.8, 30.6	15			
	*alexandrinus*	Tuzla (36 | G)	36.7, 35.1	25			
	*alexandrinus*	Al Wathba (37 | H)	24.3, 54.6	25			
	*alexandrinus*	Lake Eton (38 | I)	49.1, 46.7	14			
	*alexandrinus*	Xinjiang (39 | J)	47.7, 87.5	7			
	*alexandrinus*	Bohai (40 | K)	39.1, 118.2	5			
Kittlitz's plover	N/A	Senegal 41 | Z)	16.4, –16.3	13		Polygamous	Resident
*Charadrius pecuarius*		Gabon (42 | Y)	–0.5, 10.0	8			
dos Remedios 2013		Kenya (43 | X)	–0.5, 36.3	28			
(African mainland)		Tanzania (44 | W)	–2.9, 35.9	2			
Eberhart‐Phillips et al. 2015		Namibia (45 | V)	–18.9, 16.4	2			
(Madagascar)		Namakia (54 | A)	–15.9, 45.8	29	587,000		
		Tsiribihina Delta (56 | B)	–19.7, 44.4	4			
		Kirindy Mite (57 | C)	–20.9, 43.9	5			
		Fanjakana (58 | D)	–21.7, 45.1	3			
		Mangoky (59 | E)	–21.7, 43.4	2			
		Morombe (60 | E)	–21.8, 43.4	2			
		Andavadoaka (61 | E)	–22.1, 43.3	28			
		Ifaty (62 | F)	–23.2, 43.6	2			
		Toliara Tsiongobory (63 | F)	–23.3, 43.6	2			
		Tsimanampetsotsa (66 | G)	–24.0, 43.7	30			
		Nosimborona (68 | G)	–25.1, 44.1	2			
Madagascar plover	N/A	Boanamary (51 | A)	–15.8, 46.3	2	11,100	Monogamous	Resident
*Charadrius thoracicus*		Mahavavy (52 | A)	–15.8, 45.8	13			
Eberhart‐Phillips et al. 2015		Marambitsy (53 | A)	–15.9, 45.7	17			
		Ankazobe (55 | B)	–17.3, 44.1	3			
		Kirindy Mite (57 | C)	–20.9 43.9	7			
		Mangoky (59 | C)	–21.7, 43.4	3			
		Andavadoaka (61 | D)	–22.1, 43.3	24			
		Ifaty (62 | E)	–23.2, 43.6	4			
		Anakao (64 | F)	–23.7, 43.7	3			
		Besambay (65 | F)	–24.0, 43.7	5			
		Tsimanampetsotsa (66 | F)	–24.0, 43.7	28			
		Andranomasy (67 | F)	–24.2, 43.7	3			
white‐fronted plover	*marginatus*	Namibia (49 | Z)	–22.6, 14.5	18		Monogamous	Resident
*Charadrius marginatus*	*marginatus*	South Africa (50 | Y)	–34.1, 18.4	11			
dos Remedios 2013 (African mainland)	*tenellus*	Marambitsy (53 | A)	–15.9, 45.7	39	206,300		
Eberhart‐Phillips et al. 2015	*tenellus*	Namikia (54 | A)	–15.9, 45.8	3			
(Madagascar)	*tenellus*	Kirindy Mite (57 | B)	–20.7, 43.9	18			
	*tenellus*	Fanjakana (58 | C)	–21.7, 45.1	3			
	*tenellus*	Andavadoaka (61 | D)	–22.1, 43.3	32			
	*tenellus*	Tsimanampetsotsa (66 | E)	–24.1, 43.8	24			
chestnut banded plover	*venustus*	Kenya (46 | A)	–1.9, 36.3	12	301,000	Monogamous	Resident
*Charadrius pallidus*	*venustus*	Tanzania (47 | A)	–2.9, 35.9	12			
Eberhart‐Phillips et al. 2015	*pallidus*	Namibia (48 | B)	–22.6, 14.5	39			

Mating system references are provided in Table S3. Information on breeding range size, mating system and migratory status are provided at species level. White‐fronted and Kittlitz's plover mainland Africa populations were used only to corroborate spatial patterns found on Madagascar where sampling was more fine scale. Breeding range size for white‐fronted plover and Kittlitz's plover refers only to Madagascar, not Africa mainland. Loc Prior = different letters correspond to different location prior groupings.

Due to potential bias of null alleles during the estimation of population subdivision (F_ST_) and genetic distance (Chapuis and Estoup 2007; Dabrowski et al. 2014), null allele frequencies and genotyping errors were estimated for all data using Microchecker 2.2.3 (Van Oosterhout et al. 2004). Loci identified as having null alleles in the majority of the populations were removed for Bayesian clustering analysis, and pairwise F_ST_ values were corrected for the presence of null alleles using Free NA (Chapius and Estoup 2007). Individuals with more than 15% missing data were excluded from further analyses.

We used a Bayesian clustering algorithm implemented in STRUCTURE 2.3.4 (Pritchard et al. 2000) to determine the extent of population structure within each species. We used the admixture model with location information as a prior, an approach that is required when structure is expected to be weak (Pritchard et al. 2000; Hubisz et al. 2009). This approach improves cluster outcomes by favoring the clustering of individuals that were sampled together. However, it is worth noting that this method does not detect structure if there is none (Pritchard et al. 2000; Hubisz et al. 2009). Location priors for each population are provided in Table 1. For breeding locations with less than 10 samples we ran the analysis twice, first giving them unique location priors and again after removing these populations. All analyses were run with a burn‐in period of 100,000 followed by 1,000,000 Monte Carlo Markov Chain (MCMC) repeats for 10 replicates. Initially, the number of clusters tested were between one and the maximum number of locations sampled (Table 1). We then summarized the results with STRUCTURE HARVESTER v 0.6.94 (Earl and VonHoldt 2012) and estimated the most likely number of clusters present based on likelihood and Delta K (Evanno et al. 2005). Bar plots representing admixture proportions for the most likely K values were examined to assess whether the results of Delta K and likelihood methods were biologically meaningful. Individual admixture proportion information was merged from the ten repeats using the “full search” method in CLUMPP v 1.1.2 (Jakobsson and Rosenberg 2007). If the initial best model suggested *K* ≥ 2 and the admixture proportions of individuals within these populations was less than 0.01, the dataset was split into the identified clusters and we repeated the Bayesian clustering until the best model in STRUCTURE was *K* = 1, similar to progressive partitioning (Hobbs et al. 2011).

The number of clusters identified by STRUCTURE were compared for species with different mating systems (“monogamous” or “polygamous”) and migratory behavior (“resident” or “migratory”). Sea distance is an effective barrier of gene flow in plovers (Küpper et al. 2012). For species distributed and sampled on more than one land mass, we included only the dataset with the largest number of samples and locations. Species were assigned to categories “one genetic cluster” or “more than one genetic clusters” and we compared frequencies to expected 1:1 values using Fisher's exact tests (Fisher 1922).

We used the scoring system based on Thomas et al. (2007) to classify the mating system of each species (Székely et al. 2004; García‐Peña et al. 2009; Olson et al. 2009) and updated the mating system information for species with new data (Tables S2 and S3). However, we simplified the scoring for the purpose of this study using only two instead of five categories, since only a few dispersers per generation are required to maintain gene flow (Spieth 1974; Mills and Allendorf 1996). We classified the categories 0 and 1 of Thomas et al. (2007), which correspond to ≤1% polygamy in either sex observed during breeding behavior studies, as “monogamous” and groups 2–4 (for species that are known to display more than 1% polygamy in either sex) as “polygamous.” Migratory status was classified as either “migrant” (including partially migrant species) or resident. Migratory information was collected from Bird Life International (http://www.birdlife.org/datazone/species, accessed: January 2017) (Tables S2 and S3).

To examine the degree of isolation‐by‐distance (IBD) for the 10 plover species we performed Mantel Tests (Mantel 1967; Mantel and Valand 1970) using a population‐based approach instead of alternative landscape genetic approaches (e.g., multiple regression analysis) since individual location and environmental data were not available for all species. We calculated Euclidean distance matrices between populations using GenALEx 6.501 (Peakall and Smouse 2012). Using log‐transformed geographic distances (Legendre and Fortin 2010) provided the same qualitative results (results not shown). We calculated pairwise Rousset's linearised F_ST_ (“F_ST_” hereafter) from the null allele corrected F_ST_ values, using the following equation: F_ST_/(1–F_ST_) (Rousset 1997). All Mantel tests were performed with the package “adegenet” (Jombart 2008).

To test whether mating system and/or migratory status affects spatial genetic patterns, we used the slope of a linear regression line between genetic (F_ST_) and geographic distance for each species as a proxy for the strength of IBD (“IBD gradient” hereafter). This was calculated because of potential bias involved in directly comparing average F_ST_ values between species due to the ascertainment biases of microsatellite markers, since 75% of the markers used were developed for one species specifically (Küpper et al. 2007).

Following tests for normality of the IBD gradient, we performed phylogenetic least squares analysis (PGLS; Freckleton et al. 2002) to account for phylogenetic autocorrelation between species using the “caper” package (Orme 2013) in addition to generalized linear models (GLM) with Gaussian errors to examine the influence of mating system and migratory behaviour on the IBD gradient using “species” as the statistical unit. The recently published *Charadrius* phylogeny (dos Remedios et al. 2015) was used to measure phylogenetic relatedness between species for the PGLS analysis. Species with large breeding range sizes are likely to have greater levels of differentiation between populations compared to those with smaller ranges (Gavrilets and Vose 2005; Losos and Parent 2009; Kisel and Barraclough 2010), therefore we incorporated breeding range size into the models. Due to large differences between species breeding range sizes, which may influence the IBD gradient, log breeding range size was included in the model. As our sample size is small (*n* = 10) we fitted and compared single parameter models to avoid overfitting of models that may lead to inflation of statistical significance (Harrell 2001). The best‐fitting model was selected using an information theoretic approach (Burnham and Anderson 2002). This method ranks the models based on Akaike information criterion corrected for small sample sizes (AICc) and we assessed support for each model based on the differences in AICc (Δ*_i_*) and Akaike weights (*w_i_*) (Burnham and Anderson 2002). Substantial support for a model is indicated by Δ*_i_*‐ values of less than two and of these, highly optimal models will have *w_i_* values of more than 0.9 (Burnham and Anderson 2002). Model selection was performed using the “MuMIn” package (Bartoń 2016).

### SUBSPECIES RICHNESS IN SHOREBIRDS

To test our hypotheses that (1) polygamy restricts diversification and (2) migration restricts diversification, we used the subspecies richness of shorebird species (Order: *Charadriidae*; suborders: *Charadrii, Chionidi, Scolopaci*, and *Thinocori*) as a proxy for the degree of diversification. This allowed us to test for drivers of diversification in a much larger dataset. Avian subspecies richness has been used as a proxy for population differentiation in previous studies testing the drivers of diversification (Belliure et al. 2000; Phillimore and Owens 2006; Martin and Tewksbury 2008). We used subspecies information from the IOC World Bird List v 7.1 (Gill and Donsker 2016). This database is updated annually with new information from peer reviewed articles. Subspecies delineations are not always supported by genetic data (Phillimore and Owens 2006), however, in absence of genetic data these delineations provide a useful proxy for diversification in comparative studies at lower taxonomic levels. We classified mating systems and migratory status using the same methods as in the plover analyses above (Tables S2 and S3). We again performed PGLS analysis and in addition to mating system and migratory status we also included log breeding range size. Shorebirds without mating system information or with only anecdotal evidence of mating system category were excluded, as were species without breeding range size data.

We selected 100 phylogenetic trees at random using the phylogeny of Jetz et al. (2012), downloaded from http://birdtree.org (accessed in: December 2016). We repeated the analysis using both Hackett et al. (2008) and Ericson et al. (2006) phylogenetic backbones and no differences were found between the methods. We removed four species (*C. nivosus, Coenocorypha huegeli, Nycticryphes semicollaris*, and *Gallinago delicata*) from the analysis as they were not included in the Jetz et al. (2012) phylogeny. This resulted in a final dataset of 136 shorebirds species (Tables S2 and S3) consisting of 109 monogamous species, 27 polygamous species or 83 migrant species and 53 resident species.

PGLS analysis was repeated for each of the 100 trees and the original model formula was as follows:

$$\begin{array}{ccc} & & \;\text{Total}\;\text{number}\;\text{of}\;\text{subspecies}\;∼\;\text{mating}\;\text{system}\;+\;\text{migratory}\;\text{status}\;\\ & & \;+\;\text{migratory}\;\text{status}\;\times \;\text{mating}\;\text{system}\;\\ & & \;+\;\text{log10}\;\text{breeding}\;\text{range}\;\text{size} \end{array}$$

We then simplified models removing the least significant variable in a stepwise manner. As with IBD gradient GLMs we assessed the model fit for all model combinations using Δ*_i_* and *w_i_* values (Burnham and Anderson 2002).

For all statistical analyses, unless stated otherwise, we used R version 3.3.2 (R Development Core Team 2016).

## Results

### GENETIC DIFFERENTIATION IN PLOVERS

**Figure 2:**
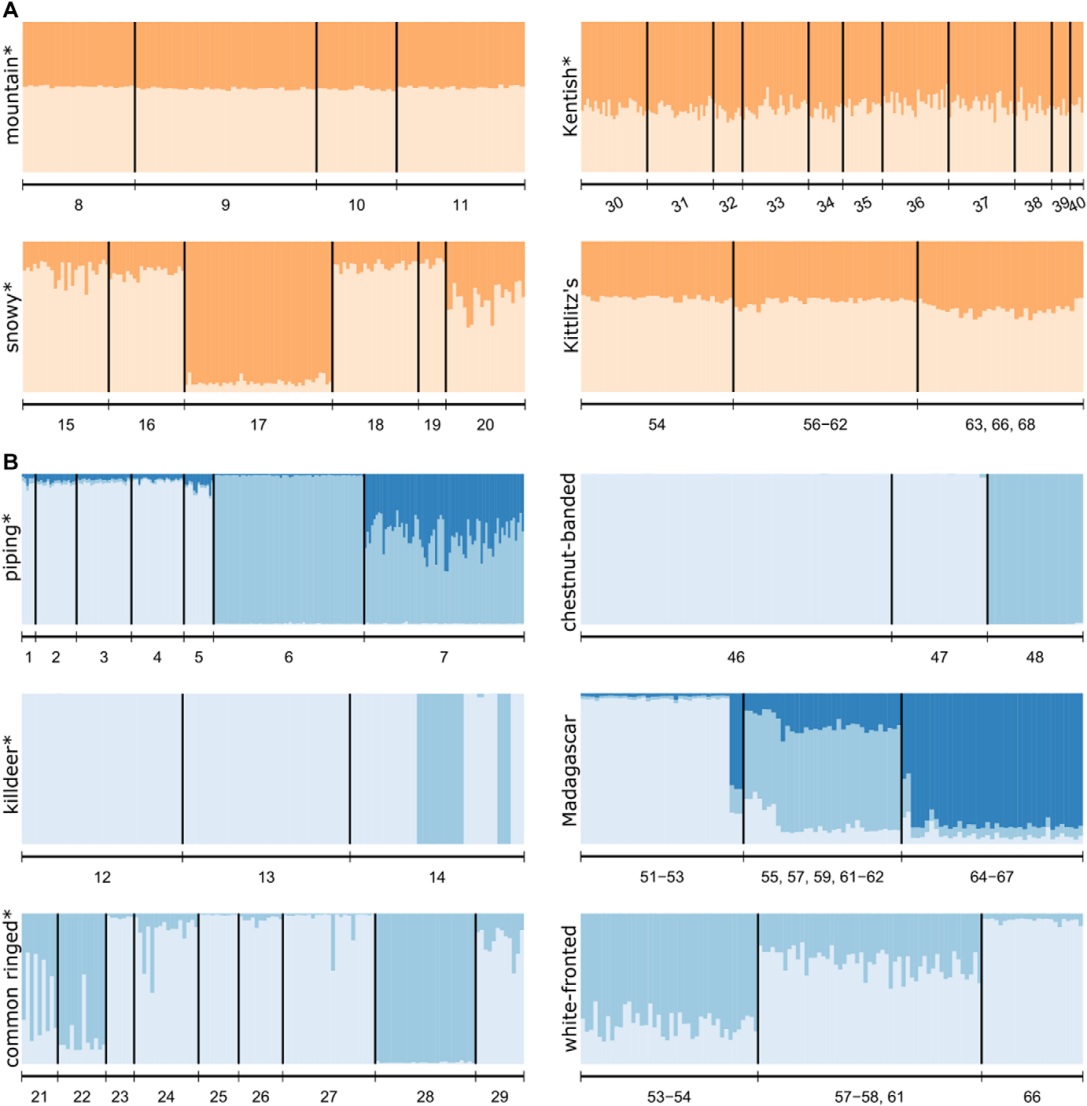
Bayesian population clustering of *Charadrius* plovers according to genetic differentiation in (A) polygamous and (B) monogamous plover species. Migratory species are indicated by asterisk, otherwise a species is an all year resident. Each vertical line represents an individual, colours represent the membership proportion to a given genetic cluster. Models with two or three clusters are presented. See table 1 for site ID number for each species.

We identified one locus, Calex14 with a high probability of having null alleles in the killdeer, this locus was excluded from further analyses in this species. The average number of alleles per locus indicated large variation in genetic diversity between species (mean = 6.4 ± 3.5 SD). No difference in the clustering outcome was found when removing populations with less than ten individuals (data available on request). Progressive partitioning increased piping plover clustering outcome from two to three, indicating that in addition to a split between the two subspecies (*C. m. circuminctus* and *C. m. melodus*), there is also differentiation in *C. m. melodus* between the Canadian and U.S. American sampling sites (Fig. 2B).

Mating system but not migratory behavior was associated with the number of genetic clusters across the 10 species (Fisher's exact tests: mating system: *P* = 0.033; migratory status: *P* = 1). We found fewer clusters within polygamous (mean ± SD: 1.25 ± 0.5) than within monogamous species (2.33 ± 0.5). We did not detect any differentiation within three of the four polygamous species across their sampled breeding populations (Fig. 2A), whereas we detected at least two genetic clusters within all six monogamous species, comprising two clusters in four species and more than two clusters in two species (Fig. 2B). The white‐fronted and Kittlitz's plover exhibited consistent patterns between Madagascar and the African mainland, that is we detected genetic structure among monogamous white‐fronted plover populations but not among polygamous Kittlitz's plover populations within each land mass. To avoid pseudoreplication, we included only the larger Madagascar dataset for both species in the subsequent analyses.

**Figure 3:**
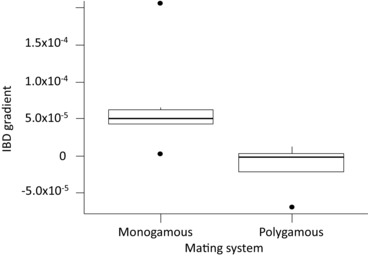
Isolation‐by‐distance gradient of monogamous (N = 6) and polygamous (N = 4) *Charadrius* plovers.

Across all plovers IBD was weak (Fig. 3 and Table 2). Three monogamous species, white‐fronted plover, piping plover, and the common ringed plover showed significant IBD (Monte Carlo test observation, *r* = 0.397, 0.749, and 0.28 respectively; *P* = 0.05, 0.02, 0.05, respectively; Table 2), whereas for all other species we did not detect a significant association. The best model to explain variation in IBD gradient among the 10 plover species contained only “mating system” as an explanatory variable (PGLS *w_i_* = 0.86) and no other model had a Δ*_i_*‐ ≤ 2. The model suggested that monogamous species have significantly higher IBD gradients than polygamous species (PGLS: df = 8, *t* = –2.49, *P* = 0.05). Neither breeding range size nor migratory status predicted IBD gradients in plovers. For full model results of the PGLS and the GLM analyses see Table S4.

**Table 2:** Patterns of isolation‐by‐distance across ten *Charadrius* plovers. Rousset's linearised F_ST_ was used as genetic distance in Mantel tests. *r* = Mantel test regression coefficient. Significant isolation‐by‐distance values (p<0.05) indicated with *

Plover species	*r*	F_ST_ gradient
**Kentish**	0.19	7.15E‐07
**Kittlitz's**	−0.28	−6.71E‐05
**mountain**	0.74	1.37E‐05
**snowy**	−0.10	−3.90E‐06
**white‐fronted**	0.40*	4.37E‐05
**Madagascar**	0.16	4.60E‐05
**piping**	0.76*	6.57E‐05
**common ringed**	0.28*	3.86E‐06
**chestnut‐banded**	0.99	2.08E‐04
**killdeer**	0.98	5.42E‐05

### SUBSPECIES RICHNESS IN SHOREBIRDS

**Figure 4:**
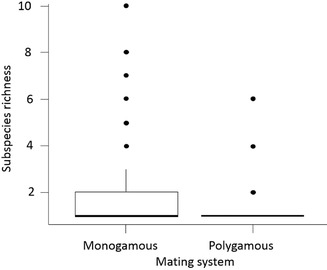
Subspecies richness of monogamous (N = 108) and polygamous (N = 28) shorebird species (Order: *Charadriidae*; suborders: *Charadrii, Chionidi, Scolopaci* and *Thinocori*).

Phylogenetic analysis in shorebirds showed that subspecies richness was best predicted by a model that included both mating system and breeding size range (Table S5). The minimal model indicated that monogamous species are divided into significantly more subspecies than polygamous species (Fig. 4) and shorebirds with larger breeding ranges harboured more subspecies than small range species (PGLS model 3: df = 133, mating system *t* = –2.26, *P* = 0.026; log breeding range size *t* = 1.98, *P* = 0.05). Consistent with genetic results in plovers, migratory behavior did not predict subspecies richness (PGLS model 2: df = 132, migratory behavior *t* = –0.165, *P* = 0.896; Table S5).

## Discussion

We investigated whether diversification in shorebirds is related to mating and/or migration behaviour using two complementary indices of population diversification: genetic differentiation in *Charadrius* plovers and subspecies richness across shorebird species. Consistent with previous studies (Møller and Cuervo 1998; Owens et al. 1999; Arnqvist et al. 2000) we found a relationship between mating system and diversification. However, contrary to previous suggestions that sexual selection facilitates speciation (West‐Eberhard 1983; Panhuis et al. 2001; Ritchie 2007) we found that polygamous shorebird species (i.e. those with higher competition for mates), showed less genetic structure, weaker isolation‐by‐distance and lower subspecies richness compared to monogamous species. These results are consistent with the “dispersal‐to‐mate” hypothesis (i.e., intense sexual selection in polygamous species promotes breeding dispersal), which in turn leads to widespread gene flow across the distribution range (Küpper et al. 2012). Our interpretations are supported by recent direct studies on breeding dispersal of polygamous sandpipers using satellite tag telemetry, where lekking male pectoral sandpipers show exceptionally long distance breeding dispersal moving up to 13,045 km during a single breeding season in search for new mating opportunities (Kempenaers and Valcu 2017). Similarly, in polygynous mammals polygynous males disperse between neighboring populations, presumably to increase their access to oestrus females (Greenwood 1980; Olupot and Waser 2001) suggesting that the dispersal of the polygamous sex is influenced by the distribution of the opposite sex (Greenwood 1980).

Using genetic data from multiple shorebirds we show the evolutionary consequences of mating behavior at the population level. Instead of promoting genetic isolation of populations, sexual selection rather seems to constrain speciation due to mate access pressure. The results on the genetic differentiation of plover populations were mirrored by our findings of subspecies richness across shorebirds that showed fewer subspecies in polygamous compared to monogamous shorebird species. Both datasets included polygynous and polyandrous taxa and hence sequentially polygamous males and females may be responsible for maintaining high gene flow. Subspecies delineations are based often largely on divergent phenotypic characters and do not necessarily reflect findings on differentiation of neutral genetic markers (Phillimore and Owens 2006). Nevertheless, in our study, we found genetic support for all subspecies delineations within the plover species analyzed (Table S6). Further, since subspecies definitions vary widely among authors and may not be supported by genetic data, subspecific delineation may in any case provide a complementary measure of ecological divergence that is then also associated with mating systems. Finally, subspecies richness may represent a more conservative measure for population differentiation than genetic differentiation since we found additional genetic structure *within* subspecies in the piping plover (*C. m. melodus*, Fig. 2B), the common ringed plover (*C. h. hiaticula* and *C. h. tundra*, Fig. 2A) and the snowy plover (*C. n. nivosus*, Fig. 2A).

Our findings contribute to the debate concerning the role of sexual selection in speciation (Gage et al. 2002; Morrow et al. 2003; Kirkpatrick and Nuismer 2004; Maan and Seehausen 2011; Servedio and Kopp 2012; Servedio and Bürger 2014; Ellis and Oakley 2016). Previous studies have suggested at least five arguments to explain why sexual selection may not appear to promote diversification. Firstly, inconsistent results may emerge if both speciation and extinction rates are elevated in sexually selected species, and these two processes counterbalance each other (Morrow et al. 2003, but see: Morrow and Fricke 2004). Secondly, different mating systems may evolve between species after speciation has occurred (e.g., some clades may be more likely to develop certain breeding behavior than others) and thus sexual selection is independent of speciation due to other mechanisms (e.g., local adaptation (Gage et al. 2002)). Thirdly, sexual selection may play a part in speciation, but mate preference alone may not be strong enough to prompt complete reproductive isolation (van Doorn et al. 2004; Servedio 2011; Servedio and Kopp 2012; Servedio and Bürger 2014). Fourthly, the effects of ecological speciation may mask the influence of sexual selection and these two forces could work antagonistically or together in speciation processes (Kraaijeveld et al. 2011; Maan and Seehausen 2011; Wagner et al. 2012). Finally, these inconsistent findings may in part be due to differences in methodologies used to investigate the relationship between sexual selection and speciation (Kraaijeveld et al. 2011).

Here, we provide a hypothesis which emphasises that dispersal driven by mate access pressure needs to be taken into account in discussions concerning the importance of sexual selection in diversification processes. According to the “dispersal‐to‐mate” hypothesis, polygamous adults (polyandrous females or polygynous males) looking for new mates may often disperse to increase their pool of potential mates. When polygamous individuals reproduce at several sites they become a major contributor to high gene flow. Field data show that male polygamous sandpipers disperse large distances during the breeding season (Kempenaers and Valcu 2017) and similarly, female polyandrous plovers tend to exhibit larger scale movements than males (Székely and Lessells 1993; Stenzel et al. 1994). These differences will ultimately be reflected in population genetic patterns. Consistent with female biased dispersal, maternally inherited mtDNA is less structured, whereas the Z‐chromosomal DNA is more structured than autosomal microsatellites in the polyandrous Kentish plover (Küpper et al. 2012). However, the latter result may also reflect typical sex‐specific natal dispersal patterns where female birds disperse more than males (Greenwood 1980; but see: Mabry et al. 2013).

Natal dispersal may chiefly serve to avoid inbreeding but it has been also been linked to the mating system (Greenwood 1980). Sex‐biased dispersal in birds and mammals may be related to either resource defence (birds) or mate defence (mammals) and hence related to mating strategies. Greenwood (1980) suggested that avian monogamy is consistent with a resource defence mating system that leads to female biased dispersal, whereas polygamy is linked to mammalian mate defence and male biased dispersal. In contrast to natal dispersal, our results imply that breeding dispersal will be dictated by the direction of polygamy, that is female biased in polyandrous population but male biased under polygyny. Two processes may explain why polygamous species have lower population divergence levels compared to monogamous species. In species with high sexual selection such as lekking species, males may either disperse to compete for additional mates, exploiting locally synchronized females (Kempenaers and Valcu 2017) or in the case of subordinate males they may disperse to find a space on a lek (Greenwood 1980). Habitat and mate availability may also be a strong factor driving female breeding dispersal in polyandrous species (Küpper et al. 2012; Cruz‐López et al. in press).

In this study, we are unable to determine the relative influence of natal versus breeding dispersal. To distinguish between the influence of natal and breeding dispersal on spatial genetic patterns, in addition to establishing whether dispersal patterns do truly differ between monogamous and polygamous species as predicted by the “dispersal‐to‐mate” hypothesis, further genetic, direct tracking, and ringing studies are necessary. For example, a direct comparison of dispersal propensity between males and females within species representing different mating systems would provide strong evidence to support or refute the “dispersal‐to‐mate” hypothesis. Despite huge recent technological advances in direct tracking (Kays et al. 2015), methodological challenges such as the weight of tags have so far constrained our ability to compare detailed movement behavior across an equivalent group of species as used in this study.

Contrary to our predictions, we found no support that annual migration influences spatial genetic patterns or subspecies richness in shorebirds. By undertaking seasonal migration, one would predict that migratory species have a higher dispersal ability than resident species and that this may promote higher gene flow between breeding populations (Winker 2000; Claramunt et al. 2012; Weeks and Claramunt 2014). A possible reason for this is that migratory species may vary in their degree of migratory connectivity. Migratory connectivity is the strength of the association between a breeding site and a wintering site. Strong migratory connectivity is when individuals from one breeding ground always migrate to the same wintering ground, whereas weak migratory connectivity reflects the mixing of populations on both breeding and wintering grounds (reviewed in: Webster et al. 2002). Strong connectivity between breeding and wintering grounds can result in genetic divergence between populations (Rundel et al. 2013); however, the degree of connectivity is highly variable between and even within species (Rundel et al. 2013, Webster et al. 2002). Therefore, the presence or absence of genetic structure and variable IBD gradients within the six migrant plover species in our plover dataset as well as the variation in subspecies richness of migratory shorebirds, may reflect different levels of migratory connectivity between species. In addition, the migratory category of this study encompasses species which vary in different aspects of migration such as distance travelled, the proportion of the population migrating and wintering habitat, all of which could have implications for breeding site genetic structure and by proxy, subspecies richness. For example, Kraaijeveld (2008) found support for habitat stability affecting subspecies richness in shorebirds with species that overwinter at unstable inland wetlands showing lower subspecies numbers than those overwintering at coastal sites, which are characterized by more stable conditions. Habitat stability might also shape patterns of breeding dispersal with plovers breeding in volatile habitats being more likely to disperse than those breeding under stable conditions. Nevertheless, a higher propensity for dispersal might enable species to reach remote, isolated locations such as oceanic islands where they subsequently evolve into new species in allopatry (Phillimore et al. 2006). The exact use of species and subspecies delineation in avian taxonomy is currently debated with disagreement about which species concept(s) are the easiest to operationalize (Sangster 2014; Barrowclough et al. 2016) and concerns about inappropriate grouping of populations (Gill 2014). We therefore decided to focus the plover analyses on continental populations only and because of the lack of similar genetic data for all shorebirds we did not evaluate subspecies delineation in the 136 shorebird species.

Present day spatial genetic patterns are the result of a multitude of past and present factors including demographic history (Excoffier 2004), habitat connectivity (Epps and Keyghobadi 2015) and range size (Phillimore et al. 2006). Although we did find that higher subspecies richness was associated with larger range sizes, supporting previous work (e.g., Salisbury et al. 2012), there was no such association within the plover dataset specifically. This is particularly interesting as two of the four polygamous species, Kentish and Kittlitz's plover, have extremely large breeding range sizes estimated at 13.6 M km^2^ and 16.4 M km^2^ (http://www.birdlife.org/datazone/species; accessed in: January 2017), respectively, yet both exhibit a distinct lack of continental genetic differentiation although their island populations are genetically differentiated (Küpper et al. 2012 and dos Remedios 2013).

Future studies are essential to further investigate the relationships between sexual selection, mate choice and breeding dispersal. New studies are needed to decouple natal and breeding sex‐biased dispersal patterns and to compare these across species representing different mating systems. To test the broader relevance of the “dispersal‐to‐mate” hypothesis it is necessary to explore the theoretical basis of how selection for high mate access promotes dispersal and the population genetic consequences of this movement. Theoretical studies have been conducted to explain sex‐biased dispersal in relation to mating systems (e.g., Kokko and Rankin 2006; Shaw and Kokko 2014), and these models provide excellent starting points for analysing mate access pressure, dispersal, and gene flow in relation to sexual selection.

In conclusion, we found that polygamous shorebirds exhibit reduced genetic differentiation compared to monogamous ones, consistently with previous studies carried out on Kentish and Malagasy plovers (Küpper et al. 2012, Eberhart‐Phillips et al. 2015). These results oppose the notion that sexual selection promotes diversification per se. On the contrary, it appears that polygamy‐usually associated with intense sexual selection–inhibits diversification in shorebirds by promoting gene flow among distant continental sites. Future studies are needed to test the robustness of this hypothesis in other taxa with variation in mating systems.

### AUTHOR CONTRIBUTIONS

C.K. and T.S. designed the study; C.K., N.d.R, K.H.M., S.Z. S.O‐M., S.H., D.B., and T.B. provided samples and/or microsatellite genotypes. C.K. and J.D.J. discussed the analysis methods and J.D.J. performed the analysis. J.D.J., C.K., M.W.B., and T.S wrote the first draft and all authors contributed the final manuscript.

Associate Editor: D. Filatov

Handling Editor: M. Noor

## Supporting information


**Table S1**. Microsatellite characteristics for all datasets.
**Table S2**. Migratory behaviour, mating system (M: monogamous; P: polygamous), subspecies richness and breeding range size of 136 shorebird species.
**Table S3**. References for mating system information of 136 shorebird species used in PGLS analysis.
**Table S4**. Single parameter PGLS (and GLM) model selection for isolation by distance gradient, df = degrees of freedom, AICc = Akaike information criterion corrected for small sample size.
**Table S5**. PGLS model simplification results to test explanatory variables on subspecies richness of 136 shorebird species.
**Table S6**. (A‐C) Pairwise F_ST_ between subspecies of (A) ringed plover, (B) chestnut banded plover and (C) white‐fronted plover.
