# Supplementary material for: Polygamy slows down population divergence in shorebirds
Source: Evolution. 2017 Apr 10;71(5):1313–26. doi: 10.1111/evo.13212 (PMC5484996; doi:10.1111/evo.13212)
Supplement: Supplementary file 1 — Table S1. Microsatellite characteristics for all datasets. Table S2. Migratory behaviour, mating system (M: monogamous; P: polygamous), subspecies richness and breeding range size of 136 shorebird species. Table S3. References for mating system information of 136 shorebird species used in PGLS analysis. Table S4. Single parameter PGLS (and GLM) model selection for isolation by distance gradient, df = degrees of freedom, AICc = Akaike information criterion corrected for small sample size. Table S5. PGLS model simplification results to test explanatory variables on subspecies richness of 136 shorebird species. Table S6. (A‐C) Pairwise FST between subspecies of (A) ringed plover, (B) chestnut banded plover and (C) white‐fronted plover. [file EVO-71-1313-s001.docx]

**Supplementary material:**

Polygamy slows down population divergence in shorebirds

Data archival location: doi:10.5061/dryad.vn77k

PCR conditions for previously unpublished datasets.

Primers were arranged in multiplexes unique to each species. Total reaction volume was 10 μL which contained 5 μL mastermix (Qiagen, Valencia, California), ~2 μM of the primer mix, and 10 ng DNA. PCR started with a 15-min activation cycle at 95°C followed by 35 cycles of 94°C for 30 s, annealing temperature for 90 s (Table S1) and 90 s at 72°C, with a final extension for 10 min at 72°C.

**Table S1**. Microsatellite characteristics for all datasets. KD: Killdeer; KT: Kentish plover; SN: snowy plover; RG: common ringed plover; KZ-M: Kittlitz’s plover (Madagascar); KZ-A: Kittlitz’s plover (continental Africa); MD: Madagascar plover; WF-M: white fronted plover (Madagascar); WF-A: white fronted plover (continental Africa); MT: mountain plover; PP: piping plover; CB: chestnut banded. Shaded cell indicates high probability of null alleles in this locus. Null alleles were removed for STRUCTURE analysis and corrected for in all other analyses using FREENA software.

|  |  |  | **Number of alleles** | | | | | | | | | | | |
| --- | --- | --- | --- | --- | --- | --- | --- | --- | --- | --- | --- | --- | --- | --- |
| **Locus** | **Reference** | **T_a_ °C** | **KD** | **KT** | **SN** | **RG** | **KZ-M** | **KZ-A** | **MD** | **WF-M** | **WF-A** | **MT** | **PP** | **CB** |
| BmaTATC371 | Rew *et al.*, 2006 | 56 | - | - | - | - | - | 11 | - | - | - | - | - | - |
| C201 | Funk *et al.*, 2007 | 56 | 17 | - | - | 18 | - | - | - | 3 | - | - | 3 | - |
| C204 | Funk *et al.*, 2007 | 56 | 12 | - | - | 9 | - | - | - | - | - | - | - | - |
| Calex01 | Küpper *et al.*, 2007 | 56 | - | 12 | 2 | - | 7 | 8 | 4 | - | 3 | - | - | - |
| Calex02 | Küpper *et al.*, 2007 | 56 | - | 18 | 3 | - | - | - | - | - | 6 | - | - | 3 |
| Calex04 | Küpper *et al.*, 2007 | 56 | - | 11 | 2 | - | - | - | - | - | 3 | - | - | 4 |
| Calex05 | Küpper *et al.*, 2007 | 56 | - | 7 | - | - | - | - | - | - | 2 | - | - | - |
| Calex06 | Küpper *et al.*, 2007 | 56 | - | - | - | - | 3 | - | 2 | 11 | - | - | - | - |
| Calex07 | Küpper *et al.*, 2007 | 56 | 5 | - | - | 15 | - | - | - | - | - | - | - | 5 |
| Calex08 | Küpper *et al.*, 2007 | 56 | 3 | 7 | - | - | - | - | - | - | 2 | - | 4 | 6 |
| Calex10 | Küpper *et al.*, 2007 | 56 | 14 | - | - | 3 | - | - | - | - | - | - | - | - |
| Calex11 | Küpper *et al.*, 2007 | 56 | - | 11 | 1 | - | - | - | - | - | 3 | - | - | - |
| Calex12 | Küpper *et al.*, 2007 | 56 | - | 10 | 4 | - | - | - | - | - | 1 | - | - | - |
| Calex13 | Küpper *et al.*, 2008 | 56 | - | - | - | - | - | - | - | - | - | - | 2 | - |
| Calex14 | Küpper *et al.*, 2007 | 56 | - | 27 | 8 | - | - | - | - | - | 6 | - | - | - |
| Calex15 | Küpper *et al.*, 2007 | 56 | - | - | - | - | - | 4 | - | - | - | - | - | - |
| Calex16 | Küpper *et al.*, 2007 | 56 | - | - | - | - | 4 | 5 | 3 | 4 | - | - | - | - |
| Calex17 | Küpper *et al.*, 2007 | 56 | 6 | - | - | 4 | - | - | - | - | - | - | - | - |
| Calex18 | Küpper *et al.*, 2007 | 62 | 4 | 12 | 2 | - | - | 5 | - | - | 4 | - | - | 4 |
| Calex19 | Küpper *et al.*, 2007 | 56 | - | 15 | 2 | - | 7 | 6 | 6 | 3 | 3 | - | - | 4 |
| Calex20 | Küpper *et al.*, 2007 | 56 | - | - | - | 8 | - | - | - | - | - | - | - | - |
| Calex22 | Küpper *et al.*, 2007 | 56 | 3 | 7 | - | - | - | - | - | - | 4 | - | - | - |
| Calex23 | Küpper *et al.*, 2007 | 56 | - | 21 | 7 | 7 | - | - | - | - | - | - | - | 4 |
| Calex24 | Küpper *et al.*, 2007 | 56 | - | 7 | 2 | - | - | - | - | - | - | - | - | - |
| Calex32 | Küpper *et al.*, 2007 | 56 | - | 10 | 4 | - | - | - | - | - | 6 | - | - | - |
| Calex33 | Küpper *et al.*, 2007 | 56 | - | - | - | - | 10 | 11 | 6 | - | - | - | - | 5 |
| Calex35 | Küpper *et al.*, 2007 | 56 | - | 25 | 6 | - | 6 | - | 6 | 8 | 6 | - | 2 | - |
| Calex36 | Küpper *et al.*, 2007 | 62 | - | - | - | - | 7 | - | 3 | 2 | - | - | - | - |
| Calex37 | Küpper *et al.*, 2007 | 59 | - | 21 | - | - | - | - | - | - | 3 | - | 5 | - |
| Calex39 | Küpper *et al.*, 2007 | 56 | - | 31 | 5 | - | - | - | - | - | 5 | - | - | 5 |
| Calex40 | Küpper *et al.*, 2007 | 55-62 | 19 | - | - | 19 | - | - | - | - | - | - | - | - |
| Calex43 | Küpper *et al.*, 2007 | 56 | 4 | 25 | 4 | - | - | - | 2 | - | 6 | - | - | - |
| Calex43b | Küpper *et al.*, 2008 |  | - | - | - | - | 2 | - | - | 9 | - | - | - | - |
| Calex45 | Küpper *et al.*, 2007 | 56 | - | 19 | - | - | 7 | 13 | 6 | 4 | 6 | - | - | 5 |
| GgaMan13 | Piertney *et al.*, 2002 | 56 | 3 | - | - | - | - | - | - | - | - | - | - | 3 |
| HrU2 | Primmer *et al.*, 1995 | 56 | - | 7 | 6 | - | - | - | - | - | 4 | - | - | - |
| Mopl2 | St. John *et al.*, 2007 | 58 | - | - | - | - | - | - | - | - | - | 3 | - | - |
| Mopl5 | St. John *et al.*, 2007 | 60 | - | - | - | - | - | - | - | - | - | 13 | - | - |
| Mopl6 | St. John *et al.*, 2007 | 58 | - | - | - | - | - | - | - | - | - | 2 | - | 2 |
| Mopl8 | St. John *et al.*, 2007 | 63 | - | - | - | - | - | - | - | - | - | 5 | - | - |
| Mopl9 | St. John *et al.*, 2007 | 58 | - | - | - | - | - | - | - | - | - | 7 | - | - |
| Mopl13 | St. John *et al.*, 2007 | 58 | - | - | - | - | - | - | - | - | - | 4 | - | - |
| Mopl15 | St. John *et al.*, 2007 | 58 | - | - | - | - | - | - | - | - | - | 3 | - | - |
| Mopl17 | St. John *et al.*, 2007 | 58 | - | - | - | - | - | - | - | - | - | 4 | - | - |
| Mopl18 | St. John *et al.*, 2007 | 58 | - | - | - | - | - | - | - | - | - | 2 | - | - |
| Mopl19 | St. John *et al.*, 2007 | 58 | - | - | - | - | - | - | - | - | - | 2 | - | - |
| Mopl21 | St. John *et al.*, 2007 | 58 | - | - | - | - | - | - | - | - | - | 4 | - | - |
| Mopl22 | St. John *et al.*, 2007 | 58 | - | - | - | - | - | - | - | - | - | 2 | - | - |
| Mopl24 | St. John *et al.*, 2007 | 55 | - | - | - | - | - | - | - | - | - | 13 | - | - |
| Mopl26 | St. John *et al.*, 2007 | 58 | 5 | - | - | - | - | - | - | - | - | 7 | - | 3 |
| PLL10 | Miller *et al.,* 2010 | 52 | - | - | - | - | - | - | - | - | - | - | 2 | - |
| PLL11 | Miller *et al.,* 2010 | 52 | - | - | - | - | - | - | - | - | - | - | 2 | - |
| PLL4 | Miller *et al.,* 2010 | 52 | - | - | - | - | - | - | - | - | - | - | 3 | - |
| Tgu04_004 | Dawson *et al.*, 2010 | 56 | 5 | - | - | - | - | - | - | - | - | - | - | 4 |
| Tgu06 | Slate *et al.*, 2007 | 56 | 10 | - | - | - | - | 7 | - | - | - | - | - | 2 |
| Average number of alleles per locus | | | 7.9 | 15.2 | 3.9 | 10.4 | 5.9 | 7.8 | 4.2 | 5.5 | 4.1 | 5.1 | 2.9 | 3.9 |
| Total number of loci | | | 14 | 20 | 15 | 8 | 9 | 9 | 9 | 8 | 18 | 14 | 8 | 15 |
| Mantel test statistic | | | 0.98 | 0.19 | -0.1 | 0.24 | -0.28 |  | 0.16 | 0.4 |  | 0.74 | 0.76 | 0.99 |
| IBD gradient | | | 5.42E-05 | 7.15E-07 | -3.90E-06 | 3.47E-06 | -6.71E-05 |  | 4.68E-05 | 4.37E-05 |  | 1.37E-05 | 6.57E-05 | 0.000208 |

**Linear regression results testing for relationship between genetic diversity and the detection of spatial patterns**

Average number of alleles Vs Mantel test statistic, estimate = -1.511, adjusted R^2^ = -0.08, p = 0.62

Average number of alleles Vs isolation by distance gradient, estimate = -17131.63, adjusted R^2^ = -0.005, p = 0.36

Number of loci Vs Mantel test statistic, estimate = 1.775, adjusted R^2^ = 0.09, p = 0.62

Number of loci Vs isolation by distance gradient, estimate = 12344.81, adjusted R^2^ = -0.079, p = 0.576

*Primer references*

Dawson, D. A., Horsburgh, G. J., Küpper, C., Stewart, I. R., Ball, A. D., Durrant, K. L., Hansson, B., Bacon, I., Bird, S., Klein, A., Krupa, A. P., Lee, J., Martίn-Gálvez, D., Simeoni, M., Smith, G., Spurgin, L. G., and Burke, T. (2010). New methods to identify conserved microsatellite loci and develop primer sets of high cross‐species utility–as demonstrated for birds. *Molecular Ecology Resources*, 10: 475-494.

Funk, W. C., Mullins, T. D., and Haig, S. M. (2007). Conservation genetics of snowy plovers (*Charadrius alexandrinus*) in the Western Hemisphere: population genetic structure and delineation of subspecies. *Conservation Genetics*, 8: 1287-1309.

Küpper, C., Horsburgh, G. J., Dawson, D. A., French-Constant, R. Székely, T., and Burke, T. (2007). Characterization of 36 polymorphic microsatellite loci in the Kentish plover (*Charadrius alexandrinus*) including two sex‐linked loci and their amplification in four other *Charadrius* species. *Molecular Ecology Notes*, 7: 35-39.

Küpper, C., Burke, T., Székely, T. and Dawson, D.A. (2008). Enhanced cross-species utility of conserved microsatellite markers in shorebirds. *BMC genomics*, 9: 502

Miller, M. P., Haig, S. M., Gratto-Trevor, C. L., & Mullins, T. D. (2010). Subspecies status and population genetic structure in Piping Plover (*Charadrius melodus*). *Auk*, 127: 57-71.

Piertney, S. B., Shorey, L., and Höglund, J. (2002). Characterization of microsatellite DNA markers in the white‐bearded manakin (*Manacus manacus*). *Molecular Ecology Notes*, 2: 504-505.

Primmer, C. R., Møller, A. P., and Ellegren, H. (1995). Resolving genetic relationships with microsatellite markers—a parentage testing system for the swallow. *Hirundo rustica. Molecular Ecology*, 4: 493-498.

Rew, M. B., Peery, M. Z., Beissinger, S. R., Berube, M., Lozier, J. D., Rubidge, E. M., & PalsbØll, P. J. (2006). Cloning and characterization of 29 tetranucleotide and two dinucleotide polymorphic microsatellite loci from the endangered marbled murrelet (*Brachyramphus marmoratus*). *Molecular Ecology Notes*, 6: 241-244.

Slate, J., Hale, M. C., and Birkhead, T. R. (2007). Simple sequence repeats in zebra finch (*Taeniopygia guttata*) expressed sequence tags: a new resource for evolutionary genetic studies of passerines. *Bmc Genomics*, 8: 52.

St. John, J., Kysela, R. F., and Oyler-McCance, S. J. (2007). Characterization of microsatellite loci isolated in Mountain Plover (*Charadrius montanus*). *Molecular Ecology Notes*, 7: 802-804.

**Table S2.** Migratory behaviour, mating system (M: monogamous; P: polygamous), subspecies richness and breeding range size of 136 shorebird species. Migratory behaviour and breeding range size information was obtained from Birdlife International (<http://www.birdlife.org/datazone/species>, accessed in: July 2016). * = updated mating system information since Thomas et al., (2007). References for mating are given in Table S3.

| **Genus** | **Species** | **Migratory** | **Mating system** | **N° subspp** | **Breeding range Km^2^** | **log10 breeding range** | **Reference** |
| --- | --- | --- | --- | --- | --- | --- | --- |
| *Actitis* | *hypoleucos* | Migrant | M | 1 | 25900000 | 7.413 | 50 |
| *Actitis* | *macularius* | Migrant | P | 1 | 13600000 | 7.134 | 34 |
| *Actophilornis* | *africanus* | Resident | P | 1 | 17000000 | 7.230 | 59 |
| *Anarhynchus* | *frontalis* | Migrant | M | 1 | 7700 | 3.886 | 58 |
| *Arenaria* | *interpres* | Migrant | M | 2 | 2620000 | 6.418 | 4 |
| *Arenaria* | *melanocephala* | Migrant | M | 1 | 83400 | 4.921 | 46 |
| *Bartramia* | *longicauda* | Migrant | M* | 1 | 3170000 | 6.501 | 16 |
| *Burhinus* | *capensis* | Resident | M | 4 | 12400000 | 7.093 | 26 |
| *Burhinus* | *grallarius* | Resident | M | 1 | 2570000 | 6.410 | 21 |
| *Burhinus* | *oedicnemus* | Migrant | M | 5 | 9150000 | 6.961 | 39 |
| *Burhinus* | *senegalensis* | Resident | M | 1 | 7040000 | 6.848 | 26 |
| *Burhinus* | *vermiculatus* | Resident | M | 2 | 5800000 | 6.763 | 52 |
| *Calidris* | *acuminata* | Migrant | P | 1 | 349000 | 5.543 | 17 |
| *Calidris* | *alba* | Migrant | P* | 2 | 1260000 | 6.100 | 5 |
| *Calidris* | *alpina* | Migrant | M | 10 | 4960000 | 6.695 | 31 |
| *Calidris* | *bairdii* | Migrant | M | 1 | 2810000 | 6.449 | 17 |
| *Calidris* | *canutus* | Migrant | M | 6 | 1600000 | 6.204 | 1 |
| *Calidris* | *fuscicollis* | Migrant | P | 1 | 419000 | 5.622 | 6 |
| *Calidris* | *himantopus* | Migrant | M | 1 | 399000 | 5.601 | 11 |
| *Calidris* | *maritima* | Migrant | M | 1 | 892000 | 5.950 | 69 |
| *Calidris* | *mauri* | Migrant | M | 1 | 310000 | 5.491 | 18 |
| *Calidris* | *melanotos* | Migrant | P | 1 | 2230000 | 6.348 | 7 |
| *Calidris* | *minuta* | Migrant | P | 1 | 1740000 | 6.241 | 48 |
| *Calidris* | *minutilla* | Migrant | M | 1 | 4970000 | 6.696 | 49 |
| *Calidris* | *ptilocnemis* | Migrant | M | 4 | 199000 | 5.299 | 51 |
| *Calidris* | *pusilla* | Migrant | M | 1 | 1220000 | 6.086 | 9 |
| *Calidris* | *ruficollis* | Migrant | M | 1 | 971000 | 5.987 | 73 |
| *Calidris* | *temminckii* | Migrant | P | 1 | 3450000 | 6.538 | 10 |
| *Calidris* | *tenuirostris* | Migrant | M | 1 | 1490000 | 6.173 | 73 |
| *Charadrius* | *alexandrinus* | Migrant | P | 4 | 13600000 | 7.134 | 55 |
| *Charadrius* | *asiaticus* | Migrant | M | 1 | 3030000 | 6.481 | 1 |
| *Charadrius* | *bicinctus* | Migrant | M | 2 | 580000 | 5.763 | 76 |
| *Charadrius* | *dubius* | Migrant | M | 3 | 19200000 | 7.283 | 1 |
| *Charadrius* | *falklandicus* | Migrant | M* | 1 | 809000 | 5.908 | 71 |
| *Charadrius* | *forbesi* | Migrant | M | 1 | 6930000 | 6.841 | 26 |
| *Charadrius* | *hiaticula* | Migrant | M | 3 | 4530000 | 6.656 | 74 |
| *Charadrius* | *marginatus* | Resident | M | 3 | 4440000 | 6.647 | 61 |
| *Charadrius* | *melodus* | Migrant | M | 2 | 221000 | 5.344 | 45 |
| *Charadrius* | *modestus* | Migrant | M* | 1 | 257000 | 5.410 | 57 |
| *Charadrius* | *montanus* | Migrant | P | 1 | 759000 | 5.880 | 33 |
| *Charadrius* | *morinellus* | Migrant | P | 1 | 276000 | 5.441 | 68 |
| *Charadrius* | *obscurus* | Migrant | M | 2 | 310 | 2.491 | 77 |
| *Charadrius* | *pallidus* | Resident | M | 2 | 301000 | 5.479 | 26 |
| *Charadrius* | *pecuarius* | Resident | P* | 1 | 16300000 | 7.212 | 56 |
| *Charadrius* | *peronii* | Resident | M* | 1 | 1270000 | 6.104 | 60 |
| *Charadrius* | *ruficapillus* | Resident | M* | 1 | 4810000 | 6.682 | 53 |
| *Charadrius* | *sanctaehelenae* | Resident | M* | 1 | 46 | 1.663 | 78 |
| *Charadrius* | *semipalmatus* | Migrant | M | 1 | 1660000 | 6.220 | 75 |
| *Charadrius* | *thoracicus* | Resident | M* | 1 | 11100 | 4.045 | 61 |
| *Charadrius* | *tricollaris* | Resident | M | 2 | 9220000 | 6.965 | 26 |
| *Charadrius* | *vociferus* | Migrant | M | 3 | 9100000 | 6.959 | 13 |
| *Charadrius* | *wilsonia* | Migrant | M* | 4 | 843000 | 5.926 | 19 |
| *Thinornis* | *novaeseelandiae* | Resident | M | 2 | 3 | 0.477 | 79 |
| *Chionis* | *albus* | Migrant | M | 1 | 27100 | 4.433 | 80 |
| *Chionis* | *minor* | Resident | M | 4 | 8600 | 3.934 | 81 |
| *Coenocorypha* | *aucklandica* | Resident | M | 1 | 680 | 2.833 | 82 |
| *Coenocorypha* | *pusilla* | Resident | M | 1 | 5 | 0.699 | 82 |
| *Cursorius* | *coromandelicus* | Resident | M* | 1 | 2390000 | 6.378 | 62 |
| *Cursorius* | *rufus* | Resident | M | 1 | 1390000 | 6.143 | 26 |
| *Cursorius* | *temminckii* | Migrant | M | 3 | 10900000 | 7.037 | 26 |
| *Elseyornis* | *melanops* | Resident | M | 1 | 7350000 | 6.866 | 73 |
| *Erythrogonys* | *cinctus* | Resident | M | 1 | 5110000 | 6.708 | 43 |
| *Calidris* | *pygmeus* | Migrant | M | 1 | 61900 | 4.792 | 24 |
| *Gallinago* | *gallinago* | Migrant | M | 2 | 20100000 | 7.303 | 1 |
| *Gallinago* | *media* | Migrant | P | 1 | 6130000 | 6.787 | 1 |
| *Gallinago* | *nigripennis* | Resident | M | 3 | 497000 | 5.696 | 47 |
| *Glareola* | *nordmanni* | Migrant | M | 1 | 1440000 | 6.158 | 1 |
| *Glareola* | *nuchalis* | Resident | M | 2 | 6190000 | 6.792 | 26 |
| *Glareola* | *pratincola* | Migrant | M | 2 | 5350000 | 6.728 | 1 |
| *Haematopus* | *bachmani* | Resident | M | 1 | 593000 | 5.773 | 70 |
| *Haematopus* | *fuliginosus* | Resident | M | 2 | 812000 | 5.910 | 73 |
| *Haematopus* | *leucopodus* | Resident | M* | 1 | 228000 | 5.358 | 66 |
| *Haematopus* | *longirostris* | Resident | M | 1 | 855000 | 5.932 | 27 |
| *Haematopus* | *moquini* | Resident | M | 1 | 140000 | 5.146 | 38 |
| *Haematopus* | *ostralegus* | Migrant | M | 4 | 2780000 | 6.444 | 35 |
| *Haematopus* | *palliatus* | Resident | M | 2 | 863000 | 5.936 | 2 |
| *Haematopus* | *unicolor* | Resident | M | 1 | 73500 | 4.866 | 83 |
| *Himantopus* | *himantopus* | Migrant | M | 1 | 56700000 | 7.754 | 1 |
| *Hydrophasianus* | *chirurgus* | Migrant | P | 1 | 6410000 | 6.807 | 29 |
| *Irediparra* | *gallinacea* | Resident | P | 1 | 2180000 | 6.338 | 44 |
| *Jacana* | *jacana* | Resident | P | 6 | 13900000 | 7.143 | 30 |
| *Jacana* | *spinosa* | Resident | P | 1 | 1040000 | 6.017 | 3 |
| *Limicola* | *falcinellus* | Migrant | M | 2 | 1010000 | 6.004 | 1 |
| *Limnodromus* | *griseus* | Migrant | M | 3 | 1650000 | 6.217 | 2 |
| *Limosa* | *fedoa* | Migrant | M | 2 | 716000 | 5.855 | 42 |
| *Limosa* | *lapponica* | Migrant | M | 4 | 1470000 | 6.167 | 1 |
| *Limosa* | *limosa* | Migrant | M | 3 | 7180000 | 6.856 | 1 |
| *Metopidius* | *indicus* | Resident | P | 1 | 2650000 | 6.423 | 8 |
| *Microparra* | *capensis* | Resident | M | 1 | 3240000 | 6.511 | 23 |
| *Numenius* | *americanus* | Migrant | M | 1 | 1820000 | 6.260 | 49 |
| *Numenius* | *arquata* | Migrant | M | 3 | 6800000 | 6.833 | 1 |
| *Numenius* | *phaeopus* | Migrant | M | 7 | 4790000 | 6.680 | 1 |
| *Numenius* | *tahitiensis* | Migrant | M | 1 | 45300 | 4.656 | 22 |
| *Pedionomus* | *torquatus* | Resident | P | 1 | 32600 | 4.513 | 40 |
| *Phalaropus* | *fulicarius* | Migrant | P | 1 | 3900000 | 6.591 | 49 |
| *Phalaropus* | *lobatus* | Migrant | P | 1 | 5110000 | 6.708 | 65 |
| *Phalaropus* | *tricolor* | Migrant | P | 1 | 3820000 | 6.582 | 20 |
| *Philomachus* | *pugnax* | Migrant | P | 1 | 8580000 | 6.933 | 1 |
| *Pluvialis* | *apricaria* | Migrant | M | 1 | 1170000 | 6.068 | 1 |
| *Pluvialis* | *dominica* | Migrant | M | 1 | 1440000 | 6.158 | 32 |
| *Pluvialis* | *fulva* | Migrant | M | 1 | 1730000 | 6.238 | 49 |
| *Pluvialis* | *squatarola* | Migrant | M | 3 | 3980000 | 6.600 | 1 |
| *Pluvianus* | *aegyptius* | Resident | M | 1 | 5980000 | 6.777 | 1 |
| *Recurvirostra* | *americana* | Migrant | M | 1 | 1390000 | 6.143 | 25 |
| *Recurvirostra* | *avosetta* | Migrant | M | 1 | 12800000 | 7.107 | 36 |
| *Rhinoptilus* | *africanus* | Resident | M | 8 | 3000000 | 6.477 | 63 |
| *Rostratula* | *benghalensis* | Resident | P | 1 | 23400000 | 7.369 | 1 |
| *Scolopax* | *minor* | Migrant | P | 1 | 1600000 | 6.204 | 15 |
| *Scolopax* | *rusticola* | Migrant | P | 1 | 15100000 | 7.179 | 84 |
| *Tringa* | *erythropus* | Migrant | M | 1 | 3720000 | 6.571 | 1 |
| *Tringa* | *flavipes* | Migrant | M | 1 | 4590000 | 6.662 | 49 |
| *Tringa* | *glareola* | Migrant | M | 1 | 15500000 | 7.190 | 1 |
| *Tringa* | *nebularia* | Migrant | M | 1 | 12100000 | 7.083 | 72 |
| *Tringa* | *ochropus* | Migrant | M | 1 | 14900000 | 7.173 | 1 |
| *Tringa* | *solitaria* | Migrant | M | 2 | 5550000 | 6.744 | 49 |
| *Tringa* | *stagnatilis* | Migrant | M | 1 | 6300000 | 6.799 | 1 |
| *Tringa* | *totanus* | Migrant | M | 6 | 19700000 | 7.294 | 1 |
| *Tryngites* | *subruficollis* | Migrant | P | 1 | 599000 | 5.777 | 12 |
| *Vanellus* | *albiceps* | Resident | M | 1 | 7540000 | 6.877 | 26 |
| *Vanellus* | *armatus* | Resident | M | 1 | 5660000 | 6.753 | 26 |
| *Vanellus* | *chilensis* | Resident | M* | 4 | 13200000 | 7.121 | 37 |
| *Vanellus* | *cinereus* | Migrant | M* | 1 | 549000 | 5.740 | 67 |
| *Vanellus* | *coronatus* | Resident | M | 3 | 6790000 | 6.832 | 26 |
| *Vanellus* | *crassirostris* | Resident | M | 2 | 3460000 | 6.539 | 26 |
| *Vanellus* | *gregarius* | Migrant | M | 1 | 1500000 | 6.176 | 1 |
| *Vanellus* | *indicus* | Resident | M | 4 | 5220000 | 6.718 | 1 |
| *Vanellus* | *lugubris* | Resident | M | 1 | 3560000 | 6.551 | 26 |
| *Vanellus* | *melanocephalus* | Resident | M | 1 | 180000 | 5.255 | 26 |
| *Vanellus* | *melanopterus* | Resident | M | 2 | 839000 | 5.924 | 26 |
| *Vanellus* | *miles* | Resident | M | 2 | 3050000 | 6.484 | 14 |
| *Vanellus* | *senegallus* | Resident | M | 2 | 11300000 | 7.053 | 26 |
| *Vanellus* | *spinosus* | Resident | M | 1 | 8170000 | 6.912 | 1 |
| *Vanellus* | *superciliosus* | Migrant | M | 1 | 752000 | 5.876 | 26 |
| *Vanellus* | *tectus* | Resident | M | 2 | 5270000 | 6.722 | 26 |
| *Vanellus* | *tricolor* | Resident | M | 1 | 5640000 | 6.751 | 43 |
| *Vanellus* | *vanellus* | Migrant | P* | 1 | 6700000 | 6.826 | 54 |

**Table S3.** References for mating system information of 136 shorebird species used in PGLS analysis.

| **Ref.** | **Author** | **Year** | **Title** | **Journal/Publisher** | **Vol** | **Pages / DOI/ web link** |
| --- | --- | --- | --- | --- | --- | --- |
| 1 | Cramp, S. and Simmons, K.E.L. | 1983 | The Birds of the Western Palearctic Vol 3 | OUP, Oxford |  |  |
| 2 | Johnsgard, P.A. | 1981 | The Plovers, Sandpipers, and Snipes of the World | University of Nebraska Press, Lincoln and London |  |  |
| 3 | Jenni, D.A. and Collier, G. | 1972 | Polyandry in the American Jacana (*Jacana spinosa*) | Auk | 89 | 743-765 |
| 4 | Nettleship, D.N. | 1973 | Breeding ecology of turnstone Arenaria interpres at Hazen Camp, Ellesmere Island, NWT | Ibis | 115 | 202-217 |
| 5 | Reneerkens, J., van Veelen, P., van der Velde, M., Luttikhuizen, P., and Piersma, T. | 2014 | Within-population variation in mating system and parental care patterns in the Sanderling (*Calidris alba*) in northeast Greenland | Auk | 131 | 235-247 |
| 6 | Parmelee, D.F., Greiner, D.W. and Graul, W.D. | 1968 | Summer schedule and breeding biology of the white-rumped sandpiper in the Central Canadian Arctic | Wilson Bulletin | 80 | 5-29 |
| 7 | Pitelka, F.A. | 1959 | Numbers, breeding schedule, and territoriality in pectoral sandpipers of northern Alaska | Condor | 61 | 233-264 |
| 8 | Art, S.H.B. | 2000 | Population structure and breeding system of the sex‐role reversed, polyandrous Bronze‐winged Jacana *Metopidius indicus* | Ibis | 142 | 93-102 |
| 9 | Gratto-Trevor, C. | 1991 | Parental care in Semipalmated Sandpipers *Calidris pusilla*: brood desertion by females | Ibis | 133 | 394-399 |
| 10 | Hilden, O. | 1975 | Breeding system of Temminck's Stint *Calidris temminckii* | Ornis Fennica | 52 | 117-146 |
| 11 | Jehl, J.R., Jr. | 1973 | Breeding biology and systematic relationships of the stilt sandpiper | Wilson Bulletin | 85 | 115-147 |
| 12 | Pitelka, F.A., Holmes, R.T. and S.F. MacLean, Jr. | 1974 | Ecology and Evolution of Social Organization in Arctic Sandpipers | American Zoologist | 14 | 185-204 |
| 13 | Lenington, S. | 1980 | Bi-parental care in killdeer: an adaptive hypothesis | Wilson Bulletin | 92 | 8-20 |
| 14 | Cardilini, A. P., Weston, M. A., Dann, P., and Sherman, C. D. | 2015 | Sharing the Load: Role Equity in the Incubation of a Monomorphic Wader, the Masked Lapwing (*Vanellus miles*) | Wilson Journal of Ornithology | 127 | 730-733 |
| 15 | Mendall, H.L. and Aldous, C.M. | 1943 | The ecology and management of the American Woodcock | Maine Cooperative Wildlife Research Unit, Orne, Maine |  |  |
| 16 | Casey, A. E., Sandercock, B. K., & Wisely, S. M. | 2011 | Genetic parentage and local population structure in the socially monogamous upland sandpiper | Condor | 113 | 119-128 |
| 17 | Myers, J.P., Hildén, O. and Tomkovich, P. | 1982 | Exotic Calidris species of the Siberian tundra | Ornis Fennica | 59 | 175-182 |
| 18 | Holmes, R.T. | 1973 | Social behaviour of breeding western sandpipers *Calidris mauri* | Ibis | 115 | 107-123 |
| 19 | Cox., L. M. | 2015 | Breeding Biology of Wilson's Plovers (*Charadrius Wilsonia*): Reproductive Success, Habitat Use, and Sex Roles | Georgia southern University, MSc thesis | 1269 | http://digitalcommons.georgiasouthern.edu/etd/1269 |
| 20 | Delechanty, D. J., Fleischer, R.C., Colwell, M. A. and Oring, L.W | 1998 | Sex-role reversal and the absence of extra-pair fertilization in Wilson's phalaropes | Animal Behaviour | 55 | 995-1002 |
| 21 | Anderson, G.J. | 1991 | The breeding biology of the bush thick-knee *Burhinus magnirostris* and notes on its distribution in the Brisbane area | Sunbird | 21 | 33-61 |
| 22 | Gill, R.E., Lanctot, R.B., Mason, J.D. and Handel, C.M. | 1991 | Observations on habitat use, breeding chronology and parental care in Bristle-thighed Curlews on the Seward Peninsula, Alaska | Wader Study Group Bulletin | 61 | 28-36 |
| 23 | Tarboton, W.R. and Fry, C.H. | 1986 | Breeding and other behaviour of the lesser jacana | Ostrich | 57 | 233-243 |
| 24 | Tomkovich, P.S. | 1995 | Breeding biology and breeding success of the spoon-billed sandpiper *Eurynorhynchus pygeus* | Russian Journal of Ornithology. In Russian with English Summary | 85 | 29-34 |
| 25 | Gibson, F. | 1971 | The breeding biology of the american avocet (*Recurvirostra americana*) in Central Oregon | Condor | 73 | 444-454 |
| 26 | Urban, E.K., Fry, C.H. and Keith, S. | 1986 | The Birds of Africa Vol II | Academic Press, London |  |  |
| 27 | Wakefield, W.C. | 1988 | Breeding resource partitioning of a mixed population of pied and sooty oystercatchers | Stilt | 13 | 39-40 |
| 28 | Barlow, M.L., Muller, P.M. and Sutton, R.R. | 1972 | Breeding data on the spur-winged plover in southland, New Zealand | Notornis | 19 | 212-249 |
| 29 | Pringle, J.D. | 1987 | The Waders of Australia | Angus and Robertson Publishers, North Ryde |  |  |
| 30 | Osborne, D.R. | 1982 | Replacement nesting and polyandry in the Wattled Jacana | Wilson Bulletin | 94 | 206-208 |
| 31 | Flodin, L. Å., and Blomqvist, D. | 2012 | Divorce and breeding dispersal in the dunlin Calidris alpina: support for the better option hypothesis? | Behaviour | 149 | 67-80 |
| 32 | Parmelee, D.F., Stephens, H.A. and R.H. Schmidt | 1967 | The birds of southereastern Victoria Island and adjacent small islands | National Museum of Canada Bulletin | 222 |  |
| 33 | Graul, W.D. | 1975 | Breeding biology of the mountain plover | Wilson Bulletin | 87 | 6-31 |
| 34 | Oring, L.W. and Lank, D.B. | 1984 | Breeding area fidelity, natal philopatry, and the social systems of sandpipers In: Eds Burger, J and Olla, BL Waders Breeding Behavior and Populations Behavior of Marine Animals Vol. 5 | Plenum Press, New York |  | 125-147 |
| 35 | Harris, M.P. | 1967 | The biology of oystercatchers *Haematopus ostralegus* on Skokholm Island, S. Wales | Ibis | 109 | 180-193 |
| 36 | Brown, P.E. | 1950 | Avocets in England | RSPB, Occasional Publication, London |  |  |
| 37 | Saracura, V., Macedo, R.H. and Blomqvist, D. | 2008 | Genetic parentage and variable social structure in breeding southern lapwings | Condor | 110 | 554-558 |
| 38 | Summers, R.W. and Cooper, J. | 1977 | The population, ecology and conservation of the black oystercatcher *Haematopus moquini* | Ostrich | 48 | 28-40 |
| 39 | Westwood, N.J. | 1983 | Breeding stone-curlews at Weeting Heath, Norfolk | British Birds | 76 | 291-304 |
| 40 | Bennett, S. | 1983 | A review of the distribution, status and biology of the plains-wanderer *Pedionomus torquatus*, Gould | Emu | 83 | 1-11 |
| 41 | Höhn, E.O. | 1975 | Notes on black-headed ducks, painted snipe, and spotted tinamous | Auk | 92 | 566-575 |
| 42 | Nowicki, T. | 1973 | A behavioral study of the Marbled Godwit in North Dakota | Central Michigan Unversity, MSc thesis |  |  |
| 43 | Marchant, S. and P.J. Higgins | 1993 | Handbook of Australian, New Zealand and Antarctic birds Vol 2 | OUP, Oxford |  |  |
| 44 | Jenni, D.A. | 1996 | Family Jacanidae (Jacanas) In: J del Hoyo, A Elliott and J Sargatal (eds), Handbook of the Birds of the World (Vol. 3 - Hoatzin to Auks) | Lynx Editions , Spain |  |  |
| 45 | Friedrich, M. J., Hunt, K. L., Catlin, D. H., and Fraser, J. D. | 2014 | The importance of site to mate choice: Mate and site fidelity in Piping Plovers | Auk | 132, | 265-276 |
| 46 | Handel and Gill | 2000 | Mate fidelity and breeding site tenacity in a monogamous sandpiper, the black turnstone | Animal Behaviour | 60 | 471-481 |
| 47 | Gichuki, C. M. | 2012 | The reproductive and foraging behaviour of the African snipe (*Gallinago nigripennis*) (Bonaparte 1839) | Ph.D Thesis Kenyatta University |  | http://ir-library.ku.ac.ke/handle/123456789/2424 |
| 48 | Hildén, O | 1978 | Occurrence and breeding biology of the little stint *Calidris minuta* in Norway | Anser, suppl, | 3 | 96-100 |
| 49 | Birds of North America *** online **** |  |  |  |  |  |
| 50 | Mee A, Whitfield DP, Thompson DBA, Burke T | 2004 | Extrapair paternity in the common sandpiper, *Actitis hypoleucos*, revealed by DNA fingerprinting | Animal Behaviour | 67 | 333-342 |
| 51 | Johnson, M., Conklin, J. R., Johnson, B. L., McCaffery, B. J., Haig, S. M., & Walters, J. R. | 2009 | Behavior and reproductive success of rock sandpipers breeding on the Yukon-Kuskokwim river delta, Alaska | The Wilson Journal of Ornithology | 121 | 328-337 |
| 52 | Kilner | 2006 | The evolution of egg colour and patterning in birds | Biological Reviews | 81 | 383-406 |
| 53 | Ekanayake KB, Weston MA, Nimmo DG, Maguire GS, Endler JA, Küpper C. | 2015 | The bright incubate at night: sexual dichromatism and adaptive incubation division in an open-nesting wader | Proceedings of the Royal Society Biological Sciences | 282 | DOI: 10.1098/rspb.2014.3026 |
| 54 | Liker, A., & Székely, T. | 1999 | Parental behaviour in the Lapwing *Vanellus vanellus* | Ibis | 141 | 608-614 |
| 55 | Székely, T. and Lessells C.M. | 1993 | Mate change by Kentish plovers *Charadrius alexandrinus* | Ornis Sand | 24 | 317-322 |
| 56 | Parra, J. E., Beltrán, M., Zefania, S., dos Remedios, N. and Székely, T. | 2014 | Experimental assessment of mating opportunities in three wader species | Animal Behaviour | 90 | 83-90 |
| 57 | St Clair JJH, Küpper C, Herrmann P, Woods RW, Székely T | 2010 | Unusual incubation sex-roles in the rufous-chested dotterel *Charadrius modestus* | Ibis | 152 | 402-404 |
| 58 | Hay, J. R. | 1984 | The behavioural ecology of the Wrybill Plover *Anarhynchus frontalis* | University of Aukland, PhD thesis |  | <http://hdl.handle.net/2292/1725> |
| 59 | Tarboton, W. R. | 1995 | Polyandry in the African jacana: the roles of male dominance and rate of clutch loss | Ostrich | 66 | 49-60 |
| 60 | Yasué, M., and Dearden, P. | 2008 | Replacement nesting and double-brooding in Malaysian Plovers *Charadrius peronii*: effects of season and food availability | Ardea | 96 | 59-72 |
| 61 | Zefania, S. and Székely, T. | 2013 | Safford and F Hawkins (eds.) The Birds of Africa Vol. 3 The Malagasy Region | Bloomsbury Publishing PLC, London | London |  |
| 62 | Munjpara, S. B. | 2013 | Ecology of the Indian *Courser Cursorius* *coromandelicus* in Abdasa, Kutch, India | Bhavnagar University, PhD thesis |  |  |
| 63 | Maclean, G.L. | 2006 | Family Glareolidae (coursers and pratincoles) In: J del Hoyo, A Elliott and J Sargatal (eds.), Handbook of the Birds of the World (Vol. 3 - Hoatzin to Auks) | Lynx Editions, Spain |  | 364-383 |
| 65 | Schamel, D., Tracy, D. M., Lank, D. B., and Westneat, D. F. | 2004 | Mate guarding, copulation strategies and paternity in the sex-role reversed, socially polyandrous red-necked phalarope *Phalaropus lobatus* | Behavioral Ecology and Sociobiology, | 57 | 110-118 |
| 66 | Newman, M. | 2013 | Brood capture by Australian pied oystercatchers | Stilt | 63 - 64 | 16-21 |
| 67 | Takahashi, M., and Ohkawara, K | 2007 | Breeding behavior and reproductive success of Grey-headed Lapwing *Vanellus cinereus* on farmland in central Japan | Ornithological science | 6 | 1-9 |
| 68 | Owens, I. P., Dixon, A., Burke, T., and Thompson, D. B. | 1995 | Strategic paternity assurance in the sex-role reversed Eurasian dotterel (*Charadrius morinellus*): behavioral and genetic evidence | Behavioral Ecology | 6 | 14-21 |
| 69 | Pierce, E. P., and Lifjeld, J. T. | 1998 | High paternity without paternity-assurance behavior in the Purple Sandpiper, a species with high paternal investment | Auk |  | 602-612 |
| 70 | Spiegel, C. S. (2008). | 2008 | Incubation patterns, parental roles, and nest survival of black oystercatchers (*Haematopus bachmani*): influences of environmental processes and potential disturbance stimuli | Oregon state university PhD thesis |  | http://hdl.handle.net/1957/10239 |
| 71 | St Clair, J. J., Herrmann, P., Woods, R. W., and Székely, T. | 2010 | Female-biased incubation and strong diel sex-roles in the Two-banded Plover *Charadrius falklandicus* | Journal of Ornithology | 151 | 811-816 |
| 72 | Thompson, P. S., and Thompson, D. B. A. | 1991 | Greenshanks *Tringa nebularia* and long‐term studies of breeding waders | Ibis | 133 | 99-112 |
| 73 | Tomkovich, P. S., and Weston, M. A. | 2007 | Breeding Ecology IN: ‘Shorebirds of Australia’(Eds A Geering, L Agnew and S Harding) | CSIRO Publishing, Clayton |  |  |
| 74 | Wallander, J, Blomqvist, D. and Lifjeld, J.T. | 2001 | Genetic and social monogamy–does it occur without mate guarding in the common ringed plover? | Ethology | 107 | 561-572 |
| 75 | Zharikov, Y., and Nol, E. | 2000 | Copulation behavior, mate guarding, and paternity in the Semipalmated Plover | Condor | 102 | 231-235 |
| 76 | Pierce, R. J. | 1989 | Breeding and social patterns of banded dotterels (*Charadrius bicinctus*) at Cass River | Notornis | 36 | 13-23 |
| 77 | Dowding, J. E., Wills, D. E., & Booth, A. M. | 1999 | Double-brooding and brood overlap by Northern New Zealand Dotterels (*Charadrius obscurus aquilonius*) | Notornis | 46 | 181-186 |
| 78 | Burns, F., McCulloch, N., dos Remedios, N., Bolton, M., & Szekely, T. | 2013 | Sex differences in incubation behaviour but not mortality risk in a threatened wader | Ibis | 155 | 877-880 |
| 79 | Davis, A. | 1994 | Breeding biology of the New Zealand shore plover *Thinornis novaeseelandiae* | Notornis | 41 | 195-208 |
| 80 | Parmelee, D. F. | 1993 | Antarctic birds: ecological and behavioural approaches | Antarctic Science | 5 | 227-232 |
| 81 | Burger, A. E. | 1979 | Breeding biology, moult and survival of Lesser Sheathbills *Chionis minor* at Marion Island | Ardea | 67 | 1-14 |
| 82 | Miskelly, C. N. | 1990 | Breeding systems of New Zealand Snipe *Coenocorypha aucklandica* and Chatham Island Snipe *C. pusilla*; are they food limited? | Ibis | 132 | 366-379 |
| 83 | Marchant, S and Higgins P.J. | 1993 | Handbook of Australian and New Zealand birds Vol. 2 Raptors to Lapwings | Oxford University Press, Melbourne |  | 748-756 |
| 84 | Hirons, G. | 1983 | A five-year study of the breeding behaviour and biology of the woodcock in England - A first report | Proc. 2nd European Woodcock and Snipe Workshop |  | 51-67 |

**Table S4**

Single parameter PGLS (and GLM) model selection for isolation by distance gradient, df = degrees of freedom, AICc = Akaike information criterion corrected for small sample size.

| Model | df | Intercept | delta | t-value | p-value | weight | AICc |
| --- | --- | --- | --- | --- | --- | --- | --- |
| **1 (mating system)** | **8** | **4.19E-05 (7.03E-05)** | **0.00 (0.00)** | **-2.49 (-2.18)** | **0.05 (0.06)** | **0.86 (0.78)** | **-138.9 (-158.3)** |
| 2 (migratory or resident) | 8 | 2.24E-05(1.59E-04) | 4.46 (3.90) | -0.99 (0.75) | 0.36 (0.48) | 0.09 (0.11) | -134.4 (-154.4) |
| 3 (log breeding range size) | 8 | 6.65E-05(2.24E-05) | 5.54 (3.98) | -0.36 (-0.79) | 0.73 (0.45) | 0.05 (0.11) | -133.3 (-154.3) |

**Table S5** PGLS model simplification results to test explanatory variables on subspecies richness of 136 shorebird species. df = degrees of freedom. AIC = Akaike information criterion.

| **Model** | **Effect** | **estimate** | | ***t*-value** | ***p*-value** | **AIC** | **df** |
| --- | --- | --- | --- | --- | --- | --- | --- |
| 1 | Mating system | -0.890 | ± 0.384 | -2.315 | 0.022 | 500.23 | 131 |
| 1 | Migratory status | -0.144 | ± 0.295 | -0.487 | 0.627 |  |  |
| 1 | Mating system : Migratory status | 0.545 | ± 0.692 | 0.787 | 0.433 |  |  |
| 1 | Log10 breeding range size | 0.211 | ± 0.113 | 1.861 | 0.065 |  |  |
|  |  |  |  |  |  |  |  |
| 2 | Mating system | -0.724 | ± 0.321 | -2.255 | 0.026 | 498.87 | 132 |
| 2 | Migratory status | -0.044 | ± 0.266 | -0.165 | 0.869 |  |  |
| 2 | Log10 breeding range size | 0.218 | ± 0.113 | 1.935 | 0.055 |  |  |
|  |  |  |  |  |  |  |  |
| 3 | **Mating system** | -0.719 | ± 0.318 | -2.258 | **0.026** | 496.90 | 133 |
| 3 | **Log10 breeding range size** | 0.221 | ± 0.111 | 1.979 | **0.050** |  |  |

**Table S6** (A-C) Pairwise F_ST_ between subspecies of (A) ringed plover, (B) chestnut banded plover and (C) white-fronted plover. Significance is indicated by * if p<0.001

(A)

|  | *hiaticula* | *tundrae* |
| --- | --- | --- |
| *hiaticula* | 0 |  |
| *tundrae* | 0.0109* | 0 |

(B)

|  | *pallidus* | *venustus* |
| --- | --- | --- |
| *pallidus* | 0 |  |
| *venustus* | 0.4157* | 0 |

(C)

|  | *marginatus* | *tenellus* |
| --- | --- | --- |
| *marginatus* | 0 |  |
| *tenellus* | 0.5648* | 0 |
